# Supplementary material for: Analysis of Viral Promoters for Transgene Expression and of the Effect of 5′-UTRs on Alternative Translational Start Sites in Chlamydomonas
Source: Genes (Basel). 2023 Apr 21;14(4):948. doi: 10.3390/genes14040948 (PMC10138193; doi:10.3390/genes14040948)
Supplement: Supplementary file 1 [file genes-14-00948-s001.zip › Figure S1.pdf]

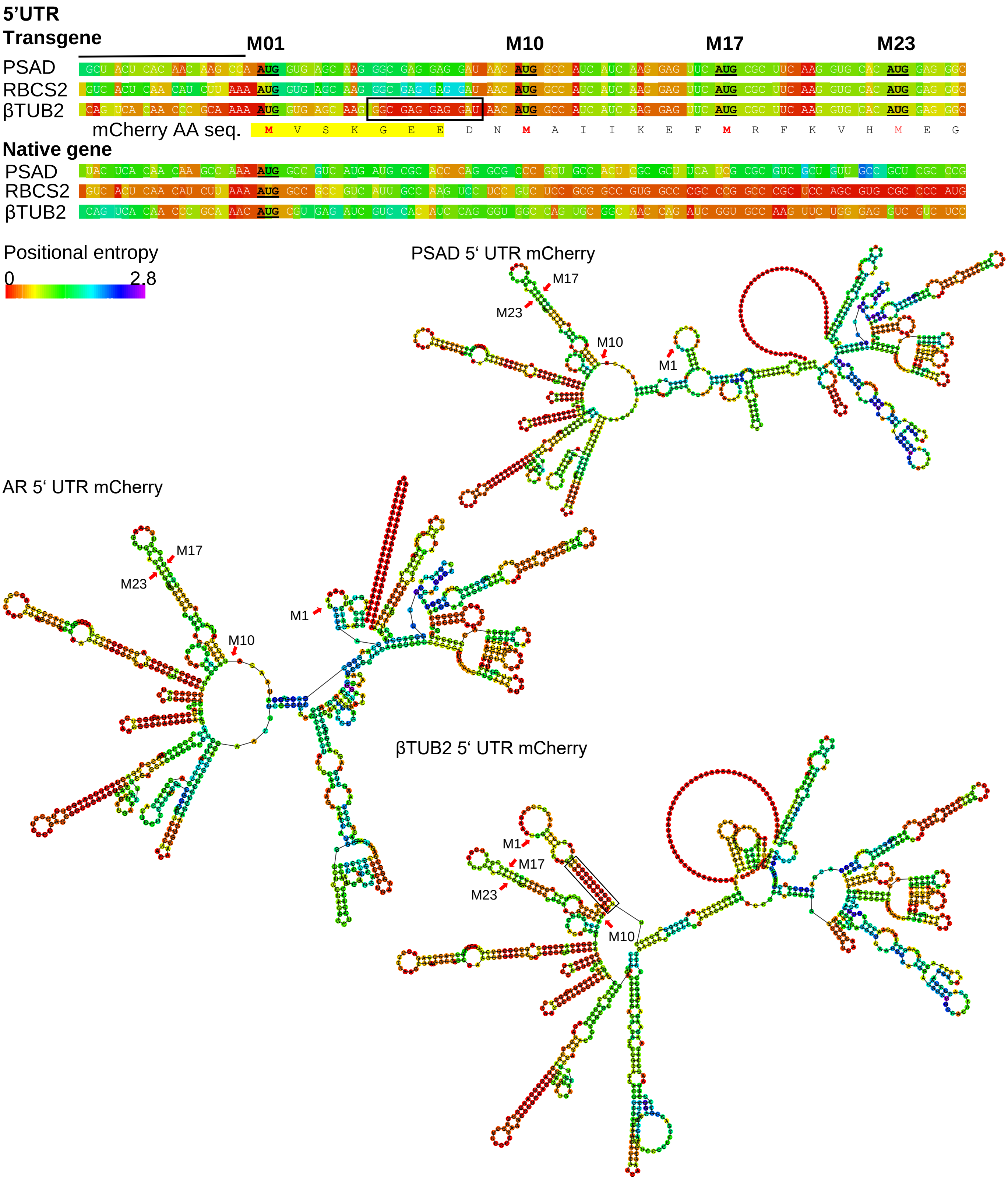

Figure S1. Prediction of mRNA secondary structures. Sequences of the transgenic *PSAD-*, *AR-*, and *AβSAP(i)-mCherry(i)* reporter transcripts and the native *PSAD*, *RBCS2*, and *βTUB2* transcripts were analyzed by the RNAfold program of the Vienna RNA package. We added a 50-nt poly-A tail after each polyadenylation signal. Shown are the positional entropies calculated by RNAfold for each nucleotide in the regions comprising 5'-UTRs and AUG start codons of the six transcripts and secondary structure predictions for the three transgene transcripts. The black box indicates a region within the *βTUB2-mCherry* transcript for which RNAfold predicts a stem-loop formed by sequences preceding the M10 AUG in the *mCherry* reporter and the *βTUB2*-5'-UTR.
