# Supplementary material for: Analysis of Viral Promoters for Transgene Expression and of the Effect of 5′-UTRs on Alternative Translational Start Sites in Chlamydomonas
Source: Genes (Basel). 2023 Apr 21;14(4):948. doi: 10.3390/genes14040948 (PMC10138193; doi:10.3390/genes14040948)
Supplement: Supplementary file 1 [file genes-14-00948-s001.zip › Supplemental Data.pdf]

Promoter sequences. MoClo fusion sites are underlined.

Viral promoters:

P_{AVCP1}

ggagGATGACGCTCTTGTCGAAGTCAGAGTACACAAACGAGTCCGGCACAAAGGATCCATAGTGCCCGTTACCCTCTTCTGTG
CCAGACATAACCACGCCCACTGGGATATGCTTCTTGTGCCACAGAATATCTGTCACCAGCGTGGTTTTGCCGGTTCCTCGCTT
ACCAATAAACACACAGGTTCGGTTGTCTCCTATAGTAGACGGGTCAAACTTTTTTAGCTTCAGGCTCATGGTGATTGCGTGAG
GATAGAGTGGTGAGCAAGAAGGCACTGCAGATGTCAGGCAAAGAGCGTTCACGTGATTCTGCTGTGCGCGGTCTGTGTGTCTG
TCTGTATGTATAGTGAAGCAAAACAAAAAATCCCAGCGCAGTTTTCCTTGCAATAGCGCGCACGTCGTTTTCTATTTAAAGTT
TGAATGGACGCTTAGGAAGAAAGAGTCGCACGTCCTCGGGGGGAGGTAATCCCCGCCACGCAGCCAACAACACaatg

P_{AVCP2}

ggagCTGGCATTCCATAAGGGTTAGGGCACACGTGTCATTGTGAGTGACCCAGTGCAGATGGATGAAAGGAATCGTCATCATG
TACAGCATCAGAAGGTGCTTGTTAGTGGCCCATGGTCCCAGCAGAACGAACACGATAAGTACCAGGTGCAGCGCCGCTATGAG
GGTTGCAGCATACAGCTGCTGCTTAACGTTTACCCTTGCCTGGCTTTCGTTGTTGTTATCCATGCCTTTTGTTTCAGCTGGGG
CGGGATAATCGTCAGATTGATGGTGTTAACGCTTTGTACCAGGTAGGTGGATGCGACGACCGAGCACGAGTGTTTAAGAGTGC
GCACATGATTTATAAGGAACTGTTACGCAACCCGAAGCGTAATGATTAAGGGTGAGAATGAGTAGTGCGACTGTTGTGCCTGT
GTATTCGTTCAGTCACACAAAATATATTGGATTGCATACGATAGGAATCACTCATAAACAAACTTCACAACaatg

P_{AVDPol}

ggagAAGCTAGACCACGTGGCGGGCCACTTCCTGGACGAGCACAAGATCGACCTCGCCATCCCGGAGATGTTCCGGCTGTTCC
AAGGAAGCGCTCGTGACCGGAGCAAGATTGCAGAGTACTGTGTGCGTGACACGGAGTTGCCGCTGAAGCTGCTGTCCAGGCTG
GCCATCCTACCGAACATGCTGGAGATGGCAAAGGTGATGCACGTCCCCATTGAATACCTCATCCCGCGAGGTCAACAGATCAA
GGACTTCTATCAGTTTCTAAAGAAGTCCCGCGAGCGTGGGTACCTTTGCCCCTGGACCTAAACTCAAACCGAAATGTAAATAT
GTTACACGTAACAACGTTGGATAGATAGCTGGTCGTTGATTCATTTTGATGAATTGATACATCGAATGTTCAAGAGGGTGATG
CGATAATGACTTAAAGACAGGGCACCTTTCAGGGTCTTAAAGACCTGACTCAGGTGGATAAACTGTGACCGCTGCACGACAGA
GGAAATTGAGCTGCAATATTAACAaatg

P_{IVCP1}

ggagTCCGCAGCGGCATGGGCCTGCGCATCAGGTGGTGGCAGCGCCACTATCGGCACCGACTCTGCCACCTCAGGAGCTGCGT
CAGCCACCACCAGCGACGCAGGTGcCTTCTTCCGGGTGGCTGTGACCTTCTTCGGGGCCGCAGCGTCTGGGGTCGTTGCTTTT
GTGGCGCCAGCCTTGCTTGTGGTGGCTCTGGGGCGTTTGGGCGCTACAGCAGCGGGGCCTGCAGGCCCTGCAGCAGCGGCAGA
TGAGGCATCATCATCCTCAGTGGGTGCGGGTGCGGCAGCCTTGGTGGCCTTCGACACGCCCCCCAGGAGCGCGCGGATGGCCA
TACTGTACTGACGGTGACTAGAATGGTGCGCGCGGGGTATCAAGATGCACCACCAGGTGGTGCGAGCCTTAAGCTCTTTGAAA
GGGGCACTCCGTGTCTCGCAACCTGCGCGACTGTCCTGTGGCGCCCAACCTGTTGAAGAGGCTTGTGGAGTGGCACACAGTAT
CAAGCaatg

P_{IVCP2}

ggagCTGCACTACATTGCGGATGTGGTGCCTGCCGATCGCCGTGAATCTGTGATGAACAAGGTCAGGGAGTTGCAGATAATTG
AGGAGCAGCTGCAGCTGCAGGCCTCGTATGTAGAGaGTCTCGGAATGTCGGTTTCAAATGTAACCTGGCACGACAACAAGGTG
TCCGTCGACGTGTCCTTTGACAAGATTGACTTCAAGAAGTTGGAAGCCCTGATCAAGACAGGCAGCAGGCCAAGCACCGTGTT
TGAAATGCAGCTGGAAGCCCTGAATGGCACACTCAGCAGTCTCACCGACATTGCGGGTGACACGTACTTGGAGGGCGACGCGG
TGGTGTACAGCGTGGAGGGCACAGACCCTGACGACCCAGACACGTACGAGCTCGTGTTTGCTGACATCACCATTTCCTACACC
ACAAGCTACTGGAAAATCAGGGACGAATGAAGCTGCGTCTGCTCAAATTCTTTTTTTCTCAAGTTAGACCATAGTCAGAGGAG
CAAAAaatg

P_{IVRPol}

ggagCGTTCGAGTCTGTTCTGCCTCCCACACCCATGAGCCTCCCCACTGGGGACGTGAGGCTGGGATCCGTCACGGTGGAGAA
CTTGGAGCGCTTCCGCGACGAGGAGGTGAGGTACATCCTCAACGCGCAGTCACCCGAGGGTGGCCCGCCAATGGCTGTGCAGC
TCACATCCCAGCGAGCCGCCTGAGAAGCCCCAGGGCAGTCCTCGAGAAAATGGTGTACAGGATCGCGACACCTGCGGTGATGG
CGTATTGCCGCACCCTACCTTTCTTGCGGGCAATGATTGCGCGCTTGCGGCGGAAGTAGGTTCCCCGCTCCAGCACCAGCAGC
GCCACGCCGCTGGCTATTAGGATCACCATGTCTGCGGGCAGCATGGTGTTGCTGTACTGAGATGCCCTGTTCTTTGGGTCTGG
CTCTCGCCGCGAAAAAGACATAAGGGCTCGGTGGGCCAGTACTTGGTAGGACACCCGGCCTCAATCGGCAGGGAATTTAGCGC
GCAACaatg

Benchmark promoters:

P$_{PSAD}$

PSAD Promoter, PSAD 5’UTR

ggagCACACACCTGCCCGTCTGCCTGACAGGAAGTGAACGCATGTCGAGGGAGGCCTCACCAATCGTCACACGAGCCCTCGTC
AGAAACACGTCTCCGCCACGCTCTCCCTCTCACGGCCGACCCCGCAGCCCTTTTGCCCTTTCCTAGGCCACCGACAGGACCCA
GGCGCTCTCAGCATGCCTCAACAACCCGTACTCGTGCCAGCGGTGCCCTTGTGCTGGTGATCGCTTGGAAGCGCATGCGATGA
CGAAGGGGCGGAGCAGGCGGCCTGGCTGTTCGAAGGGCTCGCCGCCAGTTCGGGTGCCTTTCTCCACGCGCGCCTCCACACCT
ACCGATGCGTGAAGGCAGGCAAATGCTCATGTTTGCCCGAACTCGGAGTCCTTAAAAAGCCGCTTCTTGTCGTCGTTCCGAGA
CATGTTAGCAGATCGCAGTGCCACCTTTCCTGACGCGCTCGGCCCCATATTCGGACGCAATTGTCATTTGTAGCACAATTGGA
GCAAATCTGGCGAGGCAGTAGGCTTTTAAGTTGCAAGGCGAGAGAGCAAAGTGGGACGCGGCGTGATTATTGGTATTTACGCG
ACGGCCCGGCGCGTTAGCGGCCCTTCCCCCAGGCCAGGGACGATTATGTATCAATATTGTTGCGTTCGGGCACTCGTGCGAGG
GCTCCTGCGGGCTGGGGAGGGGGATCTGGGAATTGGAGGTACGACCGAGATGGCTTGCTCGGGGGGAGGTTTCCTCGCCGAGC
AAGCCAGGGTTAGGTGTTGCGCTCTTGACTCGTTGTGCATTCTAGGACCCCACTGCTACTCACAACAAGCCaatg

P$_{AR}$

HSP70A Promoter, RBCS2 Promoter, RBCS2 5’UTR

ggagGAAGGGCCGCGACGGTTCGAGAACCGACTTGAGGGCGCCAAACGAGCCCGAGCCGCCGTTGCGCCAGGCGAAACCAGAA
CCGTAGATTAATGCACTTGAGCTATTCATTGGAGCGATCTGCCGGGGACAGCGGGTCTGGCGTGCGCGCGATTGGAGATCGCA
AATTACATATGTCTGCGTGACGGCGGGGAGCTCGCTGAGGCTTGACATGATTGGTGCGTATGTTTGTATGAAGCTACAGGACT
GATTTGGCGGGCTATGAGGGCGGGGGAAGCTCTGGAAGGGCCGCGATGGGGCGCGCGGCGTCCAGAAGGCGCCATACGGCCCG
CTGGCGGCACCCATCCGGTATAAAAGCCCGCGACCCCGAACGGTGACCTCCACTTTCAGCGACAAACGAGCACTTATACATAC
GCGACTATTCTGCCGCTATACATAACCACTCAGCTAGCGATCCCGGGCGCGCCAGAAGGAGCGCAGCCAAACCAGGATGATGT
TTGATGGGGTATTTGAGCACTTGCAACCCTTATCCGGAAGCCCCCTGGCCCACAAAGGCTAGGCGCCAATGCAAGCAGTTCGC
ATGCAGCCCCTGGAGCGGTGCCCTCCTGATAAACCGGCCAGGGGGCCTATGTTCTTTACTTTTTTACAAGAGAAGTCACTCAA
CATCTTAAaatg

P$_{A\beta SAP(i)}$

HSP70A Promoter, βTUB2 Promoter, βTUB2 5’ UTR, RBCS2i1, engineered ATANTT motifs are underlined

ggagGCTGAGGCTTGACATGATTGGTGCGTATGTTTGTATGAAGCTACAGGACTGATTTGGCGGGCTATGAGGGCGGGGGAAG
CTCTGGAAGGGCCGCGATGGGGCGCGCGGCGTCCAGAAGGCGCCATACGGCCCGCTGGCGGCACCCATCCGGTATAAAAGCCC
GCGACCCCGAACGGTGACCTCCACTTTCAGCGACAAACGAGCACTTATACATACGCGACTATTCTGCCGCTATACATAACCAC
TCAGCTAGCTTAAGATCCCATCACCGGTCTGGCACTTTCTTGCGCTATGACACTTCCAGCAAAAGGTAGGGCGGGCTGCGAGA
CGGCTTCCCGGCGCTGCATGCAACACCGATGATACTTATGCTTCGACCCCCCGAAGCTCCTTCGGGGCTGCATGGGCGCTCCG
ATGCCGCTCCAGGGCGAGCGCTGTTTAAATAGCCAGGCCCCCGACTGCAAAGACATTATAGCGAGCTACCAAAGCCATACTTC
AAACACCTAGATCACTACCACTTCTACACAGGCCACTCGAGCTTGTGGTGAGTCGACGAGCAAGCCCGGCGGATCAGGCAGCG
TGCTTGCAGATTTGACTTGCAACGCCCGCATTGTGTCGACGAAGGCTTTTGGCTCCTCTGTCGCTGTCTCAAGCAGCATCTAA
CCCTGCGTCGCCGTTTCCATTTGCAGATCGCACTCCGCTAAGGGGGCGCCTCTTCCTCTTCGTTTCAGTCACAACCCGCAAaa
tg
